# The Extended Utility of CHA_2_DS_2_VASc and HAS-BLED Scores in the Selection for Transcutaneous Left Atrial Appendage Closure

**DOI:** 10.3390/jcm9113438

**Published:** 2020-10-26

**Authors:** Witold Streb, Katarzyna Mitręga, Stanisław Morawski, Wiktoria Kowalska, Zbigniew Kalarus

**Affiliations:** Department of Cardiology, Congenital Heart Diseases and Electrotherapy SUM, Silesian Center for Heart Diseases, 41-800 Zabrze, Poland; k.mitrega@sccs.pl (K.M.); s.morawski@sccs.pl (S.M.); vicky.kowalska@gmail.com (W.K.); karzab@sum.edu.pl (Z.K.)

**Keywords:** atrial fibrillation, stroke, atrial appendage, prognosis, left atrial appendage closure

## Abstract

Background and purpose: Left atrial appendage closure (LAAC) is an option for stroke prevention in atrial fibrillation patients. Randomized studies have demonstrated the effectiveness and safety of LAAC but included patients with an average risk of stroke and bleeding complications. The current study aimed to assess the extended utility of CHA_2_DS_2_VASc (congestive heart failure; hypertension; age ≥75 years [doubled]; type 2 diabetes; previous stroke, transient ischemic attack, or thromboembolism [doubled]; vascular disease; age 65 to 75 years; and sex category) and HAS-BLED (hypertension; abnormal renal/liver function; stroke; bleeding history or predisposition; labile INR, elderly, drugs/alcohol concomitantly) scores for qualification and prognosis after LAAC. Methods: The study population comprised 270 patients aged 72.8 ± 8.78 years. The occluders used were the Amplatzer Amulet (*N* = 205), Amplatzer Cardiac Plug (*N* = 53), and Watchman device (*N* = 12). The prognosis after LAAC was analyzed for different cohorts of patients distinguished based on different CHA_2_DS_2_VASc and HAS-BLED scores. The mean duration of follow-up was 21.6 ± 10.3 months. Results: The observed rates of ischemic stroke and bleeding were much lower than that expected (2.2% vs. 5.6%, and 0.76% vs. 6.05%, respectively). The mortality rate did not differ concerning the CHA_2_DS_2_CVASc score. It was significantly lower (8.3%) for HAS-BLED < 3, and it raised to 17.9% for HAS-BLED = 3 and to 25.9% for HAS-BLED > 3. Significant differences (*p* = 0.003) occurred for Kaplan–Meier curves for extreme HAS-BLED subgroups. A composite endpoint was most often found in high/very high risk of bleeding patients. Conclusions: HAS-BLED, but not CHA_2_DS_2_CVASc score, may be a useful tool to predict the prognosis of patients after LAAC. Qualification for LAAC based on the risk of stroke should not differ from qualification for anticoagulation. Despite the worse prognosis of patients with the highest bleeding risk, this group is likely to experience the greatest benefit from reducing the bleeding risk from LAAC.

## 1. Introduction

Effective prevention of stroke in patients with atrial fibrillation remains one of the main problems in modern cardiology. The number of patients with atrial fibrillation and the number of patients requiring stroke prevention is expected to increase in the coming decades. Consequently, it is essential to select patients at risk and to choose the right anti-stroke prevention strategy. A commonly used tool to assess the risk of stroke in atrial fibrillation is the CHA_2_DS_2_VASc score. It has been shown that the categorization of patients based on the CHA_2_DS_2_VASc score to the low-risk group corresponds to a small number of thromboembolic complications observed during the year (100 person-years rate is 0.78, CI 0.58 to 1.04). In turn, categorization to an intermediate-risk (score = 1) or high-risk (score = 2) group is associated with an increase of that rate to 2.01 (CI 1.70–2.36) and 3.71 (CI 3.36–4.09), respectively [1].

The 2020 European Society of Cardiology (ESC) guidelines recommend using stroke prevention in patients with atrial fibrillation in the high-risk group. They also suggest considering that approach if the risk of stroke is estimated to be moderate [2]. The primary strategy of such prevention is non-vitamin-K oral anticoagulants (NOACs), which are preferred over vitamin K antagonists (VKAs). 

However, only some patients eligible for the prophylactic use of anticoagulants receive NOACs or VKAs. The GLORIA registry showed that only 55.2% of patients in Asia and up to 90.1% in Europe were treated with anticoagulants [3]. The number of patients for whom such prophylaxis is waived is high. A common problem is also the discontinuation of anticoagulant therapy by patients themselves. Beyer-Westendorf et al. showed that the rate of persistence with rivaroxaban therapy was only 81.5% [4]. Moreover, the patients who switched from VKAs to rivaroxaban had a similar persistence rate to subjects receiving NOACs as the first stroke prevention option. The results of other studies also confirm low adherence to stroke prevention recommendations. Martinez et al. assessed the proportion of patients remaining on NOACs or VKAs after treatment initiation and found that persistence with oral anticoagulants declined over 12 months to 63.6% for VKAs and 79.2% for NOACs [5].

In some cases, the withdrawal or interruption of the stroke prevention regimen is due to therapy complications. The cause may also be contraindications for the use of anticoagulants. The exponent of both is often severe bleeding. Therefore, it is recommended that the risk of severe bleeding should be estimated before initiation of anticoagulation therapy. Several scales of bleeding risk during anticoagulant therapy have been developed, and one of the most commonly used is the HAS-BLED (hypertension; abnormal renal/liver function; stroke; bleeding history or predisposition; labile INR, elderly, drugs/alcohol concomitantly) score; HAS-BLED ≥ 3 indicates a high risk of bleeding while using anticoagulants.

Left atrial appendage occlusion (LAAC) is an optional form of stroke prevention in patients with non-valvar atrial fibrillation. According to ESC recommendations, LAAC can be considered in patients with long-term contraindications for anticoagulant treatment [2]. However, in practice, LAAC is usually limited to patients at the highest risk of strokes, bleeding, and multiple, severe comorbidities because of potential procedural complications. Moreover, in some countries, LACC treatments are financed only for patients with both a HAS-BLED score ≥ 3 and a CHA_2_DS_2_VASc score ≥ 3, who are at high risk of both ischemic stroke and bleeding. Meanwhile, randomized studies that determined the effectiveness and safety of LAAC treatments compared to VKAs involved patients with an average risk of stroke and hemorrhagic complications who could receive anticoagulant treatment [6,7].

The current study aimed to assess the extended utility of CHA_2_DS_2_VASc and HAS-BLED scores for qualification for LAAC and post-procedural prognosis. Therefore, we compared the procedural safety and long-term effectiveness of LAAC in different patient cohorts based on stroke and bleeding risk.

## 2. Methods

The study population comprised 270 patients (117 male, 153 female) aged 72.8 ± 8.78 years who underwent LAAC procedures. The included patients had permanent (*N* = 205; 75.93%), paroxysmal (*N* = 64; 23.7%), or persistent (*N* = 1; 0.37%) atrial fibrillation, and indications of stroke prophylaxis. The mean CHA_2_DS_2_VASc score was 4.17 ± 1.47 (median 4.0), and the mean HAS-BLED score was 3.0 ± 0.75 (median 3.0). The comorbidities present in the studied population are listed in Table 1. Most of the patients had a history of severe bleeding (*N* = 177; 65.6%). In the majority of patients, there was a history of gastrointestinal tract bleeding, accounting for 35.5% of all cases.

The occluders used for implantations were the Amplatzer Amulet (*N* = 205), Amplatzer Cardiac Plug (*N* = 53), and Watchman device (*N* = 12). The procedures were performed in deep sedation and monitored by the combination of fluoroscopy with transesophageal or intracardiac echocardiography. The patents did not continue anticoagulant therapy independently of the device used. Post LAAC, the patients received dual antiplatelet therapy consisting of 75 mg ASA and 75 mg of clopidogrel for a period of 6 to 12 weeks after the procedure. The mean duration of follow-up was 21.6 ± 10.3 months. Data collection in the follow-up observation included ischemic stroke, hemorrhagic complications, and deaths for any reason. The composite endpoint of both stroke and hemorrhagic complications and a composite endpoint of stroke, hemorrhagic complications, and death for any reason were later analyzed. Survival curves obtained for subgroups of patients differing in respect to CHA_2_DS_2_VASc and HAS-BLED scores were later compared.

The event-free survival curves estimated for specific patient cohorts were compared using a log-rank test. In this study, a *p*-value < 0.05 was considered to be statistically significant. Jamovi statistical software version 1.2.17 was used for the analyses.

## 3. Results

As many as 61.6% of patients undergoing LAAC were individuals at very high risk of stroke (CHA_2_DS_2_CVASc score >3). An additional 26.1% of patients had moderate–high stroke risk (CHA_2_DS_2_CVASc score 3), and the remaining 12.3% of patients were at low–moderate stroke risk (CHA_2_DS_2_CVASc score 1 or 2). The majority (57.5%) of patients were at a very high risk of bleeding, as indicated by HAS-BLED score = 3. HAS-BLED score was assessed to be <3 in 22.4% of patients, whereas 20.1% of subjects exceeding the HAS-BLED score of 3 were at risk of bleeding. More than three-quarters of patients had both CHA_2_DS_2_CVASc and HAS-BLED scores of >3. 

**Figure 1:**
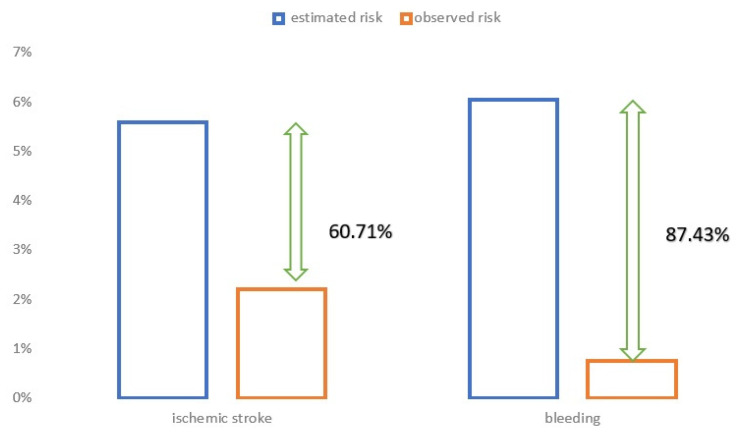
Comparison of observed versus estimated risk of ischemic stroke and bleeding complications in the studied population.

In the overall study population, we observed a significant reduction in the rate of observed incidents of ischemic strokes and bleeding complications when compared to the risk estimated based on the CHA_2_DS_2_CVASc and HAS-BLED scales (Figure 1). The percentage of patients who experienced an ischemic stroke during the follow-up time reached 2.2%, whereas the estimated risk was 5.6%. Similarly, the observed bleeding rate was much lower than expected (0.76% versus 6.05%). During the 30-month observation, 47 subjects expired (mortality rate 17.41%). Of these, the cause of death was a fatal stroke only in one case, and cardiac death was documented in just three patients. Other deaths resulted from non-cardiac conditions, which is not a surprise considering numerous and severe concomitant diseases.

**Figure 2:**
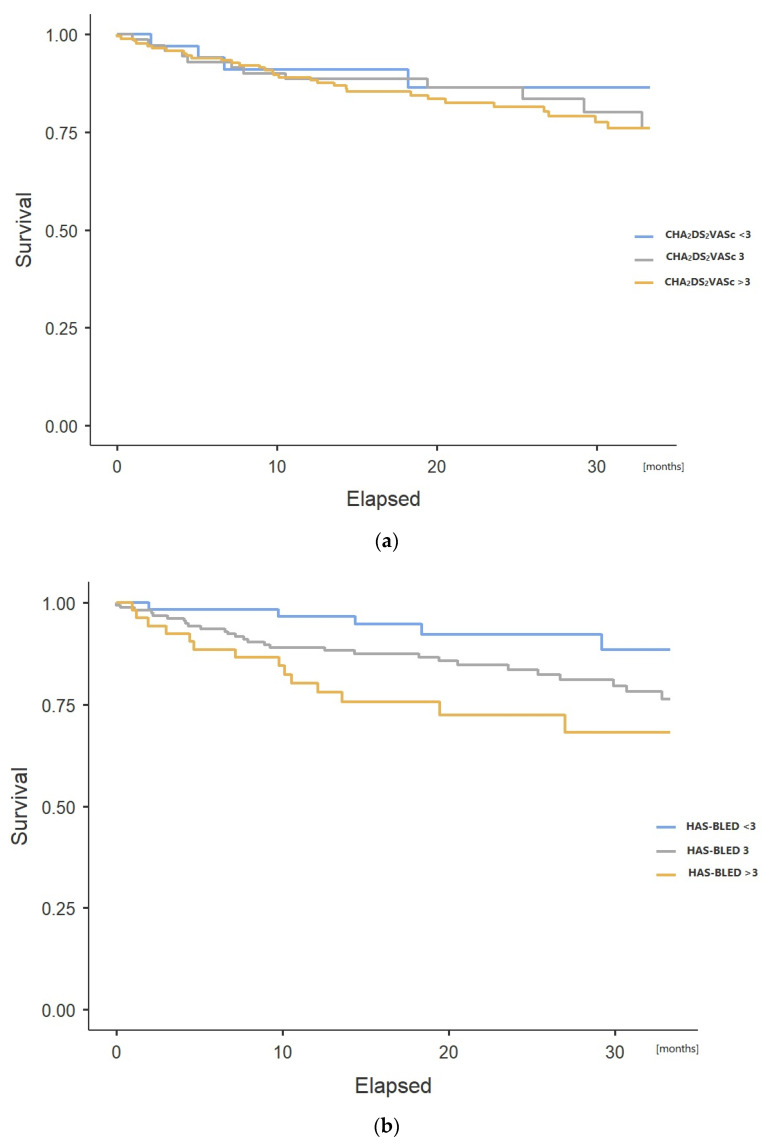
Kaplan–Meier probability of death in subgroups split according to different CHA_2_DS_2_CVASc (congestive heart failure; hypertension; age ≥75 years [doubled]; type 2 diabetes; previous stroke, transient ischemic attack, or thromboembolism [doubled]; vascular disease; age 65 to 75 years; and sex category) (**a**) and HAS-BLED (hypertension; abnormal renal/liver function; stroke; bleeding history or predisposition; labile INR, elderly, drugs/alcohol concomitantly) scores (**b**).

The mortality in patients with CHA_2_DS_2_CVASc score < 3 was 12.1%. When the CHA_2_DS_2_CVASc score was 3, the mortality was 17.1%, and for CHA_2_DS_2_CVASc > 3, it was 18.6%. A comparison of survival curves free of death estimated for groups at different stroke risk based on the CHA_2_DS_2_CVASc score did not show that patients with a higher risk of stroke had a worse prognosis (Figure 2a). The comparison of survival curves free of ischemic stroke, hemorrhage, composite endpoint, and composite endpoint including death also did not reveal significant differences in cohorts distinguished based on different CHA_2_DS_2_CVASc scores (Table 2).

More considerable differences in survival free of death were observed in subgroups distinguished based on the HAS-BLED score (Figure 2b). In the group of patients with a moderate (<3) risk of bleeding on the HAS-BLED scale, the mortality rate was significantly lower than in the other subgroups and reached 8.3%. The mortality rate was observed to be more than double (17.9%) in a subset of patients who achieved 3 scores on the HAS-BLED scale. In the subgroup of patients characterized by HAS-BLED score >3, the mortality rate was the highest (25.9%). The log-rank test showed that statistically significant differences (*p* = 0.005) occurred for survival curves free of death for extreme subgroups when the HAS-BLED score was <3 or exceeded 3. Differences in the survival curves for the HAS-BLED groups were not due to differences in the incidence of stroke or bleeding complications. There were also no other differences for the curves free from the composite endpoint, including cerebral stroke and hemorrhagic complications. Such differences were found only for the endpoint, including death from any cause (Table 2). As with mortality, the survival curves for patients with HAS-BLED < 3 also differed from the rest of the study population for the composite endpoint of stroke, bleeding complications, or death from any cause (Table 3).

## 4. Discussion

Few randomized studies have been conducted to assess the safety and effectiveness of LAAC procedures in patients with atrial fibrillation. The studies that have been conducted involved only patients eligible for oral therapy with anticoagulants. PROTECT-AF and the PREVAIL study, conducted in such a population, showed that LAAC is a non-inferior method of stroke prophylaxis compared to treatment with warfarin. The non-inferiority of the Watchman device to warfarin was proven for the primary efficacy endpoint of stroke, cardiovascular death, or systemic thromboembolism. The event rates of primary efficacy endpoints were 3% and 4.9% for Watchman and warfarin groups, respectively. The mean CHADS_2_ score was 2.3 ± 1.2 and 2.1 ± 1.2 in warfarin and device groups, respectively [7,8]. The 5-year outcomes of the PREVAIL trial, combined with the 5-year outcomes of the PROTECT AF trial, demonstrated that LAAC with Watchman provides stroke prevention in nonvalvular atrial fibrillation comparable to warfarin, with additional reductions in major bleeding, particularly hemorrhagic stroke, and mortality. Similar to the PROTECT-AF study, patients participating in that trial did not differ significantly in stroke risk in the device and warfarin group. Differences in hemorrhagic stroke, disabling/fatal stroke, cardiovascular/unexplained death, all-cause death, and post-procedure bleeding favored LAAC (hazard ratio (HR): 0.20; *p* = 0.0022; HR: 0.45; *p* = 0.03; HR: 0.59; *p* = 0.027; HR: 0.73; *p* = 0.035; HR: 0.48; *p* = 0.0003, respectively) [9]. Although both studies concluded that the benefits of LAAC treatments were associated with a lower risk of hemorrhagic complications, the lack of information about the bleeding risk in the initial characteristics in both arms of these studies is associated with ambiguous conclusions.

The observational studies also indicate the benefits of reducing the number of hemorrhagic complications after LAAC procedures. The Evolution registry collected data from 1025 patients who were eligible for LAAC based on current ESC recommendations; 15.1% of patients had a previous hemorrhagic stroke, 320 (31.3%) had a history of major bleeding, and 750 (73%) were deemed unsuitable for oral anticoagulation therapy. LAAC procedures were performed with Watchman occluders. Post-procedurally, patients received dual or single antiplatelet therapy, warfarin, or none of these drugs [10,11]. The actual major bleeding rate, excluding procedural bleeding events, was reduced by 48% compared to the calculated risk based on the HAS-BLED score. The observed stroke rate was surprisingly even further reduced than that estimated by CHA_2_DS_2_CVASc (1.1% versus 7.2%, respectively, reduction by 84%). The recently published real-world data from the National Cardiovascular Data Registry show that LAAC procedures are performed almost exclusively in the sickest patients. The mean patient age was 76.1 ± 8.1 years, the mean CHA_2_DS_2_VASc score was 4.6 ± 1.5, and the mean HAS-BLED score was 3.0 ± 1.1 [12].

In this study, the LAAC population had a higher risk of stroke and a higher risk of bleeding than those who participated in randomized studies comparing LAAC with oral anticoagulant therapy. Despite this, the results showed a significant reduction in both strokes and bleeding in long-term observations. Moreover, our results confirm that expected benefits are associated with a reduction in stroke risk and a disproportionately higher reduction in the risk of bleeding. 

The results of the prospective global Amplatzer Amulet observational study recently published by Tarantini et al. also did not show that a higher CHA_2_DS_2_CVASc score was associated with a worse prognosis of LAAC patients in terms of the incidence of stroke and transient ischemic attacks [13]. The study compared two groups of bleeding risk with the criterion of having HAS-BLED scores ≤3 and >3 points. There was a smaller reduction in the risk of bleeding in the group of patients with HAS-BLED > 3 (risk reduction by 11% vs. 9%), and after excluding cases with a history of gastrointestinal bleeding, the risk of bleeding was lower than expected when HAS-BLED was >3 (reduction of 39% vs. 44%). However, it should be noted that the HAS-BLED ≤ 3 criterion includes both patients with moderate and high bleeding risk, which could have influenced the authors’ inconclusive and relatively small difference in the reduction of bleeding risk in both subgroups. 

Our results are only partially convergent, as they do not indicate significant differences in the incidence of stroke and bleeding and worse prognosis of patients with higher CHA_2_DS_2_CVASc score. On the other hand, using a different criterion for distinguishing subgroups depending on the HAS-BLED score demonstrated the added value of the HAS-BLED scale used to assess the prognosis of patients undergoing LAAC. A HAS-BLED score ≥3 is associated with a worse prognosis, which is not due to the occurrence of more bleeding. It should be noted that patients with a lower HAS-BLED score had a significantly more frequent history of intracranial hemorrhage, while chronic diseases such as renal failure and uncontrolled blood pressure were less frequent. At the same time, the interpretation of the results shows that although HAS-BLED allows the prognosis of patients after LAAC to be assessed, it is not a tool that enables the proper determination of the bleeding risk after this procedure.

## 5. Conclusions

The HAS-BLED scale, but not CHA2DS2CVASc, may be a useful tool to predict patient prognosis after LAAC procedures. Qualification for LAAC based on stroke risk should not differ from qualification for anticoagulation, as the benefits of LAAC do not depend on the initial CHA_2_DS_2_CVASc score. Despite the worse prognosis of patients with the highest bleeding risk, this group is likely to experience the greatest benefit from reducing the bleeding risk from LAAC.

## 6. Study Limitation

The study was not a randomized study to directly compare the effects of treatment using LAAC and anticoagulants. Due to the small number of patients, the study also could not clearly show that patients with a moderate risk of stroke (CHA_2_DS_2_CVASc = 1) benefit the same as other patients.

## Figures and Tables

**Table 1:** Comorbidities in the study population.

	CHA_2_DS_2_CVASc				HAS-BLED				
	≤2	3	>3	χ^2^ *p*-Value	<3	3	>3	χ^2^ *p*-Value	Total (%)
	*N* = 33	*N* = 70	*N* = 165		*N* = 60	*N* = 165	*N* = 54		
	(% of Group)	(% of Group)	(% of Group)		(% of Group)	(% of Group)	(% of Group)		
Chronic heart failure	5	12	47	5.06	10	37	17	3.44	64
	(15.2%)	(17.1%)	(28.5%)	0.08	(16.7%)	(22.4%)	(31.5%)	0.179	(23.7 %)
Controlled hypertension	16	42	107	3.21	27	102	36	8.97	167
	(48.5%)	(60.0%)	(64.9%)	0.201	(45.0%)	(61.8%)	(66.7%)	0.011	(61.9%)
Uncontrolled hypertension	6	11	38	1.74	12	30	13	0.53	55
	(18.2%)	(15.7%)	(23.0%)	0.419	(20.0%)	(18.2%)	(20.0%)	0.767	(20.4%)
Ischemic stroke	0	7	56	27.2	5	36	22	16.6	63
	(0.0%)	(10.0%)	(33.9%)	<0.001	(8.3%)	(21.8%)	(13.3%)	<0.001	(23.3%)
Diabetes	4	15	68	15.9	15	54	18	2.02	87
	(12.1%)	(21.4%)	(41.2%)	<0.001	(25.0%)	(32.7%)	(33.3%)	0.365	(32.2%)
Chronic kidney diseases	4	12	74	22.8	14	51	25	9.41	90
	(12.1%)	(17.1%)	(44.8%)	<0.01	(23.3%)	[30.9%]	(46.3%)	0.009	(33.3%)
stage 3a	2	10	44	9.57	9	35	12	1.63	56
	(6.1%)	(14.2%)	(26.7%)	0.008	(15.0%)	(21.2%)	(22.2%)	0.442	(20.7%)
Stage 3b	1	1	15	5.55	2	8	7	5.24	17
	(3.0%)	(1.4%)	(9.1%)	0.062	(3.3%)	(4.8%)	(4.2%)	0.073	(6.3%)
stage 4	0	0	11	7.16	3	6	2	0.161	11
	(0.0%)	(0.0%)	(6.7%)	0.028	(5.0%)	(3.6%)	(3.7%)	0.923	(4.1%)
stage 5	1	1	4	0.33	0	2	4	8.56	6
	(3.0%)	(1.4%)	(2.4)	0.848	(0.0%)	(1.2%)	(7.4%)	0.014	(2.2%)
History of major bleeding	24	43	110	1.35	42	102	33	1.01	177
	(72.7%)	(61.4%)	(66.7%)	0.509	(70.0%)	(61.8%)	(61.1%)	0.604	(65.6%)
Lower gastrointestinal tract	4	14	44	3.8	11	39	12	1.22	62
	(12.1%)	([20.0%)	(26.7%)	0.150	(13.3%)	(23.6%)	(22.2%)	0.544	(23.0%)
Upper gastrointestinal tract	6	7	21	4.96	7	19	8	1.6	34
	(18.2%)	(10.0%)	(12.7%)	0.292	(11.7%)	(11.5%)	(14.8%)	0.808	(12.6%)
Intracranial hemorrhage	5	9	17	0.786	12	12	7	6.42	31
	(15.2%)	(12.9%)	(10.3%)	0.675	(20.0%)	(7.3%)	(12.9%)	0.040	(11.5%)
Urinary tract	4	5	8	2.55	5	11	1	2.4	17
	(12.1%)	(7.1%)	(4.8%)	0.279	(8.3%)	(6.7%)	(1.9%)	0.301	(6.3%)
Nasopharynx	1	2	7	4.01	1	7	2	1.44	10
	(3.0%)	(2.9%)	(4.2%)	0.405	(1.7%)	(4.2%)	(3.7%)	0.837	(3.7%)
Intraocular	2	2	3	1.97	3	3	1	1.73	7
	(3.3%)	(2.9%)	(1.8%)	0.374	(5.0%)	(1.8%)	(1.9%)	0.420	(2.6%)
Other	2	4	10	035	3	11	2	0.196	16
	(6.1%)	(5.7%)	(6.1%)	0.839	(5.0%)	(6.7%)	(3.7%)	0.906	(5.9%)

**Table 2:** Analysis of differences for survival curves calculated for different HAS-BLED and CHA_2_DS_2_CVASc scores (log-rank test).

	Survival Free of Ischemic Stroke	Survival Free of Hemorrhage	Survival Free of the Composite Endpoint	Survival Free of Composite Endpoint Including Death for Any Reason
HAS-BLED	χ^2^ = 2.42, *p* = 0.298	χ^2^ = 0.644, *p* = 0.725	χ^2^ = 0.828, *p* = 0.661	χ^2^ = 8.41, *p* = 0.015
CHA_2_DS_2_CVASc	χ^2^ = 4.06, *p* = 0.131	χ^2^ = 2.67, *p* = 0.268	χ^2^ = 0.918, *p* = 0.632	χ^2^ = 1.86, *p* = 0.395

**Table 3:** The log-rank test resulted in subgroups split according to different HAS-BLED values for the composite endpoint, including death for any reason.

	ν	*SE*	z	*p*
HAS-BLED < 3 vs. HAS-BLED = 3	6.13	2.80	2.187	0.029
HAS-BLED < 3 vs. HAS-BLED > 3	6.58	2.20	2.985	0.003
HAS-BLED = 3 vs. HAS-BLED > 3	3.83	2.92	1.310	0.190
